# Supplementary material for: Global expansion of tropical cyclone precipitation footprint
Source: Nat Commun. 2024 Jun 6;15:4824. doi: 10.1038/s41467-024-49115-1 (PMC11156673; doi:10.1038/s41467-024-49115-1)
Supplement: Supplementary file 1 — Supplementary Information [file 41467_2024_49115_MOESM1_ESM.pdf]

Supplementary Information for
Global expansion of tropical cyclone precipitation footprint

Lianjie Qin[1], Laiyin Zhu[2,*], Baoyin Liu[3], Zixuan Li[4], Yugang Tian[5], Gordon Mitchell[6], Shifei Shen[1], Wei Xu[7,8,9,*], Jianguo Chen[1,*]

[1]School of Safety Science, Tsinghua University, Beijing, 100084, China.
[2]School of Environment, Geography, and Sustainability, Western Michigan University, Kalamazoo, MI, 49008, USA.
[3]Institutes of Science and Development, Chinese Academy of Sciences, Beijing, 100190, China.
[4]School of Finance, Nankai University, Tianjin, 300350, China.
[5]School of Geography and Information Engineering, China University of Geosciences, Wuhan, 430074, China.
[6]School of Geography and Water@leeds, University of Leeds, Leeds, LS2 9JT, UK.
[7]Key Laboratory of Environmental Change and Natural Disaster of Ministry of Education, Faculty of Geographical Science, Beijing Normal University, Beijing, 100875, China.
[8]State Key Laboratory of Earth Surface Processes and Resource Ecology, Beijing Normal University, Beijing, 100875, China.
[9]Academy of Disaster Reduction and Emergency Management, Ministry of Emergency Management and Ministry of Education, Beijing Normal University, Beijing, 100875, China.
Correspondence to:
L. Zhu, laiyin.zhu@wmich.edu;
W. Xu, xuwei@bnu.edu.cn;
J. Chen, chenjianguo@tsinghua.edu.cn
L. Qin and L. Zhu contributed equally to this work.

Table of Contents

- **Supplementary Tables**
- **Supplementary Figures**

Supplementary Tables

Supplementary Table 1. The details of the total impact area and DIST30 increased area.

	Total area ($\times 10^7$ km^2)	DIST30 increased area ($\times 10^7$ km^2)	Total boundary area ($\times 10^7$ km^2)	DIST30 increased boundary area ($\times 10^7$ km^2)
Global	14.6836	8.7908 (59.8681%)	4.7317	2.5562 (54.0237%)
WPAC	3.3944	2.2027 (64.8916%)	1.1856	0.7332 (61.8389%)
NAT	2.9514	1.9168 (64.9461%)	1.0167	0.4986 (49.0452%)
EPAC	2.0687	1.0583 (51.1597%)	0.3700	0.1877 (50.7206%)
NIN	1.0067	0.3760 (37.3518%)	0.5116	0.1674 (32.7112%)
SPAC	2.2938	1.5597 (67.9981%)	0.7763	0.4933 (63.5459%)
SIN	2.9686	1.6772 (56.4975%)	0.8714	0.4761 (54.6316%)

Supplementary Table 2. The details of the areas of magnitude of the differences in DIST30 between the late period (2001-2020) and the early period (1980-1999).

	Area of DeV (×10^6 km^2)	Area of DeIV (×10^6 km^2)	Area of DeIII (×10^6 km^2)	Area of DeII (×10^6 km^2)	Area of DeI (×10^6 km^2)	Area of InI (×10^6 km^2)	Area of InII (×10^6 km^2)	Area of InIII (×10^6 km^2)	Area of InIV (×10^6 km^2)	Area of InV (×10^6 km^2)
Global	4.44 (3.03%)	5.94 (4.05%)	7.88 (5.36%)	14.89 (10.14%)	25.77 (17.55%)	30.60 (20.84%)	24.92 (16.97%)	14.75 (10.04%)	6.77 (4.61%)	10.87 (7.41%)
WPAC	0.90 (2.65%)	0.97 (2.85%)	0.90 (2.65%)	2.44 (7.18%)	6.71 (19.78%)	8.53 (25.13%)	6.20 (18.27%)	3.69 (10.87%)	1.06 (3.13%)	2.54 (7.49%)
NAT	0.78 (2.64%)	1.23 (4.16%)	2.00 (6.78%)	2.71 (9.19%)	3.63 (12.28%)	6.26 (21.22%)	6.22 (21.09%)	2.61 (8.85%)	1.90 (6.43%)	2.71 (7.36%)
EPAC	0.38 (1.82%)	0.75 (3.64%)	1.49 (7.19%)	2.40 (11.60%)	5.09 (24.59%)	5.09 (24.62%)	1.88 (9.07%)	2.29 (11.08%)	0.76 (3.69%)	0.56 (2.69%)
NIN	1.13 (11.26%)	0.57 (5.70%)	1.15 (11.43%)	1.93 (19.13%)	1.52 (15.14%)	1.31 (12.98%)	0.75 (7.42%)	0.94 (9.32%)	0.20 (1.96%)	0.57 (5.66%)
SPAC	0.53 (2.32%)	1.30 (5.67%)	0.56 (2.43%)	1.41 (6.16%)	3.54 (15.43%)	3.92 (17.08%)	5.40 (23.53%)	2.02 (8.83%)	1.40 (6.11%)	2.86 (12.46%)
SIN	0.72 (2.44%)	1.12 (3.77%)	1.78 (6.00%)	4.01 (13.50%)	5.28 (17.80%)	5.49 (18.49%)	4.47 (15.07%)	3.19 (10.74%)	1.44 (4.86%)	2.17 (7.33%)

Note: DeV means ΔDIST30 is less than -100 km; DeIV means ΔDIST30 ranges from -100 km to -75 km; DeIII means ΔDIST30 ranges from -75 km to -50 km; DeII means ΔDIST30 ranges from -50 km to -25 km; DeI means ΔDIST30 ranges from -25 km to 0; InI means ΔDIST30 ranges from 0 to 25 km; InII means ΔDIST30 ranges from 25 km to 50 km; InIII means ΔDIST30 ranges from 50 km to 75 km; InIV means ΔDIST30 ranges from 75 km to 100 km; InV means ΔDIST30 is greater than 100 km; ΔDIST30 means the difference in DIST30 between the late and early periods.

Supplementary Table 3. Abbreviated list of key variables.

**DIST30**	km	Distance between tropical cyclone center and extreme rainfall (at the threshold of 30 mm per 3hr)
**DIST50**	km	Distance between tropical cyclone center and extreme rainfall (at the threshold of 50 mm per 3hr)
**MM**	/	Month
**LAT**	°N	Latitude
**LON**	°E	Longitude
**PRES**	hPa (=mb)	Minimum pressure near the tropical cyclone center
**VMAX**	knots	Maximum sustained wind speed
**W10M**	m/s	10m wind speed
**U10M**	m/s	10m u component of wind
**V10M**	m/s	10m v component of wind
**SST**	K	Sea surface temperature
**RSST**	K	Relative sea surface temperature
**TCWV**	kg/m2	Total column water vapour
**T2M**	K	2m temperature
**RT2M**	K	Relative 2m temperature
**WS**	m/s	Wind shear

Supplementary Table 4. Abbreviated list of climate indexes.

**AAO**	Antarctic Oscillation (https://www.cpc.ncep.noaa.gov/products/precip/CWlink/daily_ao_index/aao/aao_index.html)
**AMM**	Atlantic Meridional Mode SST (https://psl.noaa.gov/data/timeseries/monthly/AMM/)
**AMO**	Atlantic Multidecadal Oscillation, unsmoothed (https://psl.noaa.gov/data/timeseries/AMO/)
**AO**	Arctic Oscillation (https://www.cpc.ncep.noaa.gov/products/precip/CWlink/daily_ao_index/ao.shtml)
**CENSO**	Bivariate ENSO Timeseries (https://psl.noaa.gov/people/cathy.smith/best/)<br>Calculated from combining a standardized SOI and a standardized Niño3.4 SST timeseries. Uses the dataset (HadISST1.1) is now used to calculate Niño 3.4 timeseries.
**EA**	Eastern Atlantic (https://www.cpc.ncep.noaa.gov/data/teledoc/ea.shtml)
**ESPI**	ENSO Precipitation Index (http://precip.gsfc.nasa.gov/ESPItable.html)
**GMSST**	Global Mean Land/Ocean Temperature<br>Note, the index is an anomaly index.
**MEIV2**	Multivariate ENSO Index (MEI V2) (https://psl.noaa.gov/enso/mei/)
**NAO**	North Atlantic Oscillation (https://www.cpc.ncep.noaa.gov/data/teledoc/nao.shtml)
**NINA1**	Niño 1+2*, Extreme Eastern Tropical Pacific SST (0-10S, 90W-80W)
**NINA3**	Niño 3*, Eastern Tropical Pacific SST (5N-5S, 150W-90W)
**NINA34**	Niño 3.4*, East Central Tropical Pacific SST (5N-5S, 170-120W)
**NINA4**	Niño 4*, Central Tropical Pacific SST (5N-5S, 160E-150W)
**ONI**	Oceanic Niño Index (https://origin.cpc.ncep.noaa.gov/products/analysis_monitoring/ensostuff/ONI_v5.php)
**PNA**	Pacific/North American (https://www.cpc.ncep.noaa.gov/data/teledoc/pna.shtml)

**PACW**	Pacific Warmpool Area Average<br>Definition: area averaged SST: 60E-170E, 15S-15N Dataset: NOAA ERSSTV5 1948-present<br>Climatology: 1981-2020
**PDO**	Pacific Decadal Oscillation (https://cicoes.uw.edu/pdo/PDO.latest) File Not Found The page you are looking for might have been removed, had its name changed, or is temporarily unavailable.
**QBO**	Quasi-Biennial Oscillation<br>Calculated at PSL (from the zonal average of the 30mb zonal wind at the equator as computed from the NCEP/NCAR Reanalysis).
**SOI**	Southern Oscillation Index<br>(https://www.cpc.ncep.noaa.gov/data/indices/Readme.index.shtml#SOICALC)
**SOLAR**	Solar Flux (10.7cm)* (http://www.spaceweather.ca/solarflux/sx-4-eng.php)
**TNA**	Tropical Northern Atlantic Index<br>Anomaly of the average of the monthly SST from 5.5N to 23.5N and 15W to 57.5W. HadISST and NOAA OI 1x1 datasets are used to create index. Climatology is 1971-2000.
**TNI**	Trans-Niño Index, Indices of El Niño Evolution<br>(https://psl.noaa.gov/gcos_wgsp/Timeseries/TNI/)
**TSA**	Tropical Southern Atlantic Index<br>Anomaly of the average of the monthly SST from Eq-20S and 10E-30W. HadISST and NOAA OI 1x1 datasets are used to create index. Climatology is 1971-2000.
**WHWP**	Western Hemisphere Warm Pool<br>(Monthly anomaly of the ocean surface area warmer than 28.5° C in the Atlantic and eastern North Pacific. Based on HadISST and NOAA OI SST (for latest value). Climatology is 1971-2000.)
**WP**	Western Pacific (https://www.cpc.ncep.noaa.gov/data/teledoc/wp.shtml)

Supplementary Figures

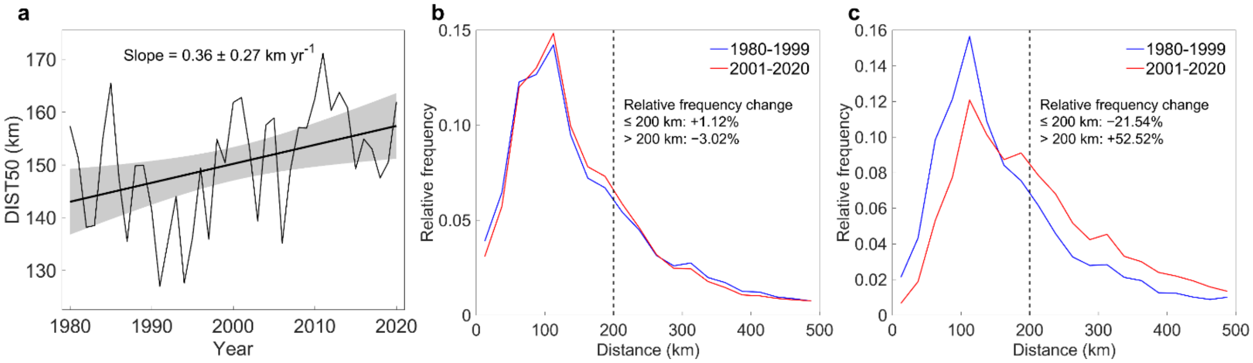

Supplementary Fig. 1. Temporal changes in DIST50 and TC rainfall. a. time series of DIST50. b, relative frequency of distance in low latitude (≤25°). c, relative frequency of distance in high latitude (>25°).

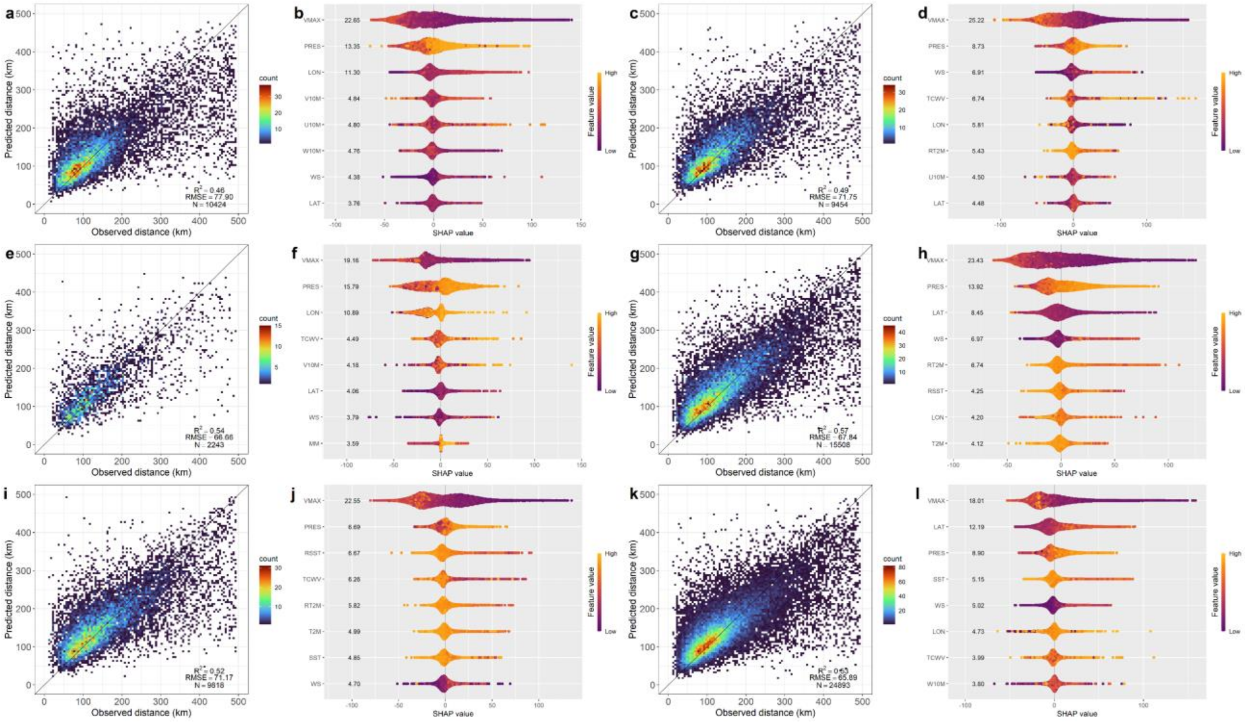

Supplementary Fig. 2. Xgboost model performance and SHAP feature importance for basin-scale DIST30. a-b, EPAC. c-d, NAT. e-f, NIN. g-h, SIN. i-j, SPAC. k-l, WPAC.

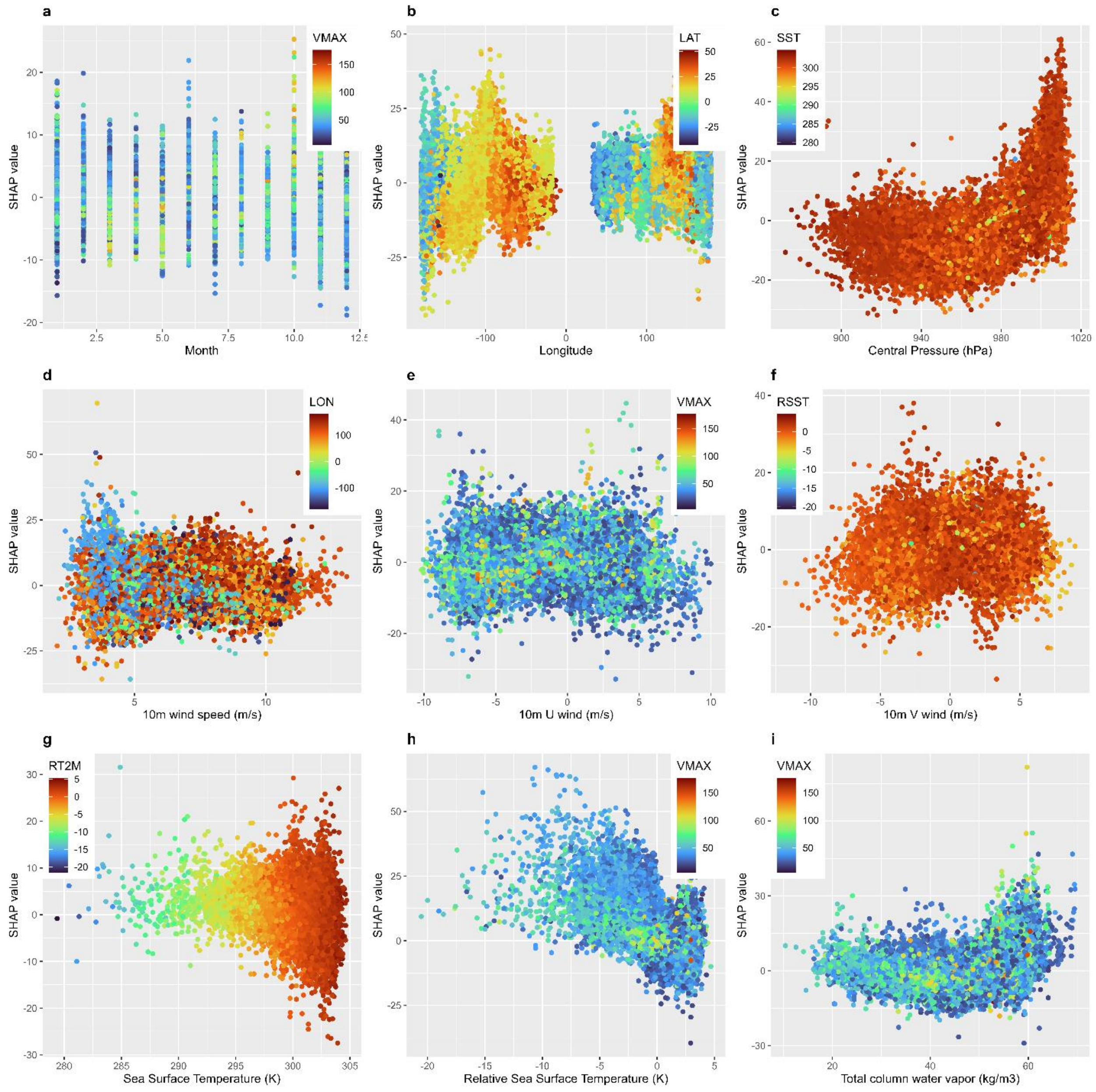

Supplementary Fig. 3. Relationship between participating features and DIST30 SHAP value for the global XGBoost model (PART 1).

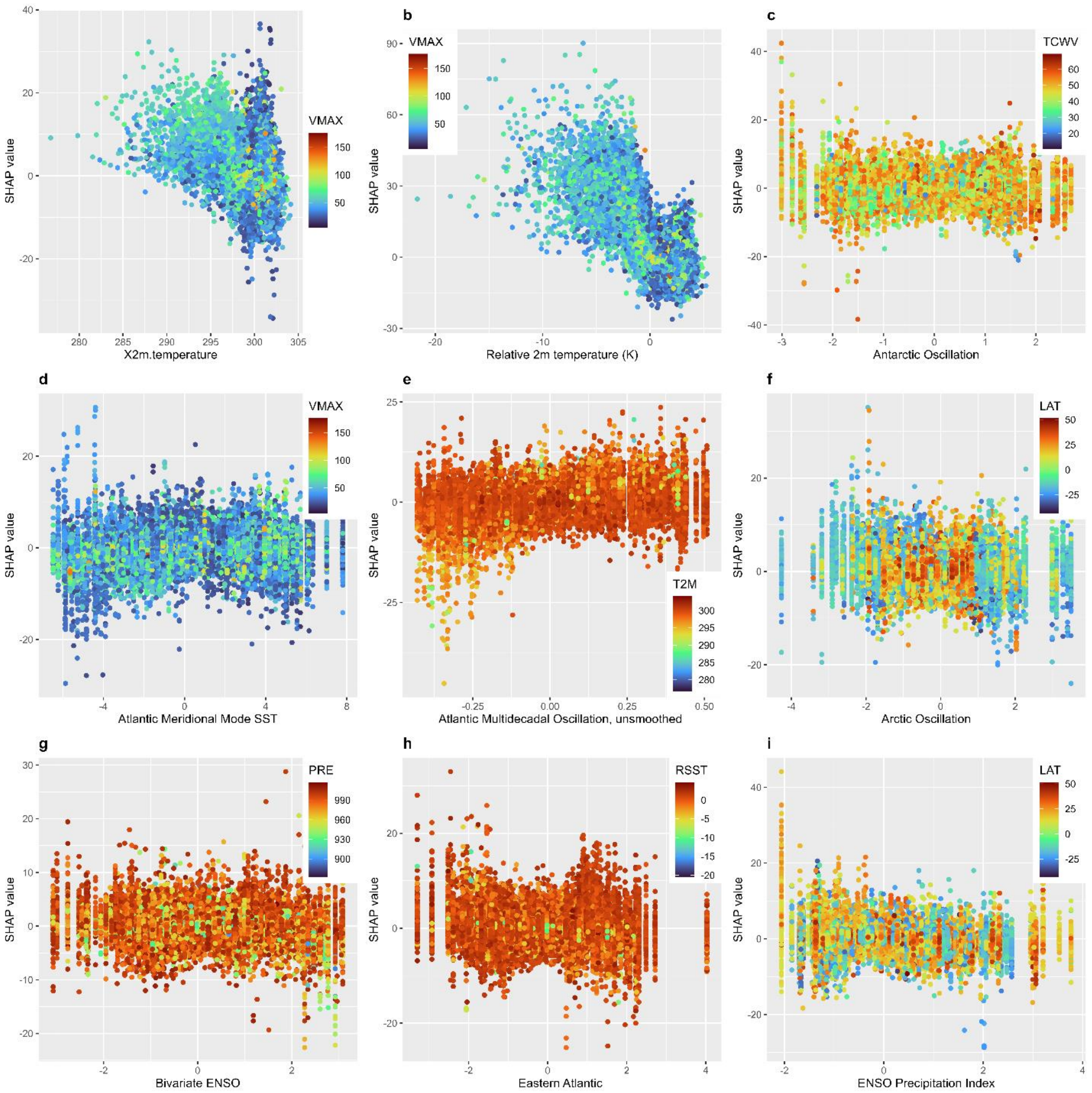

Supplementary Fig. 4. Relationship between participating features and DIST30 SHAP value for the global XGBoost model (PART 2).

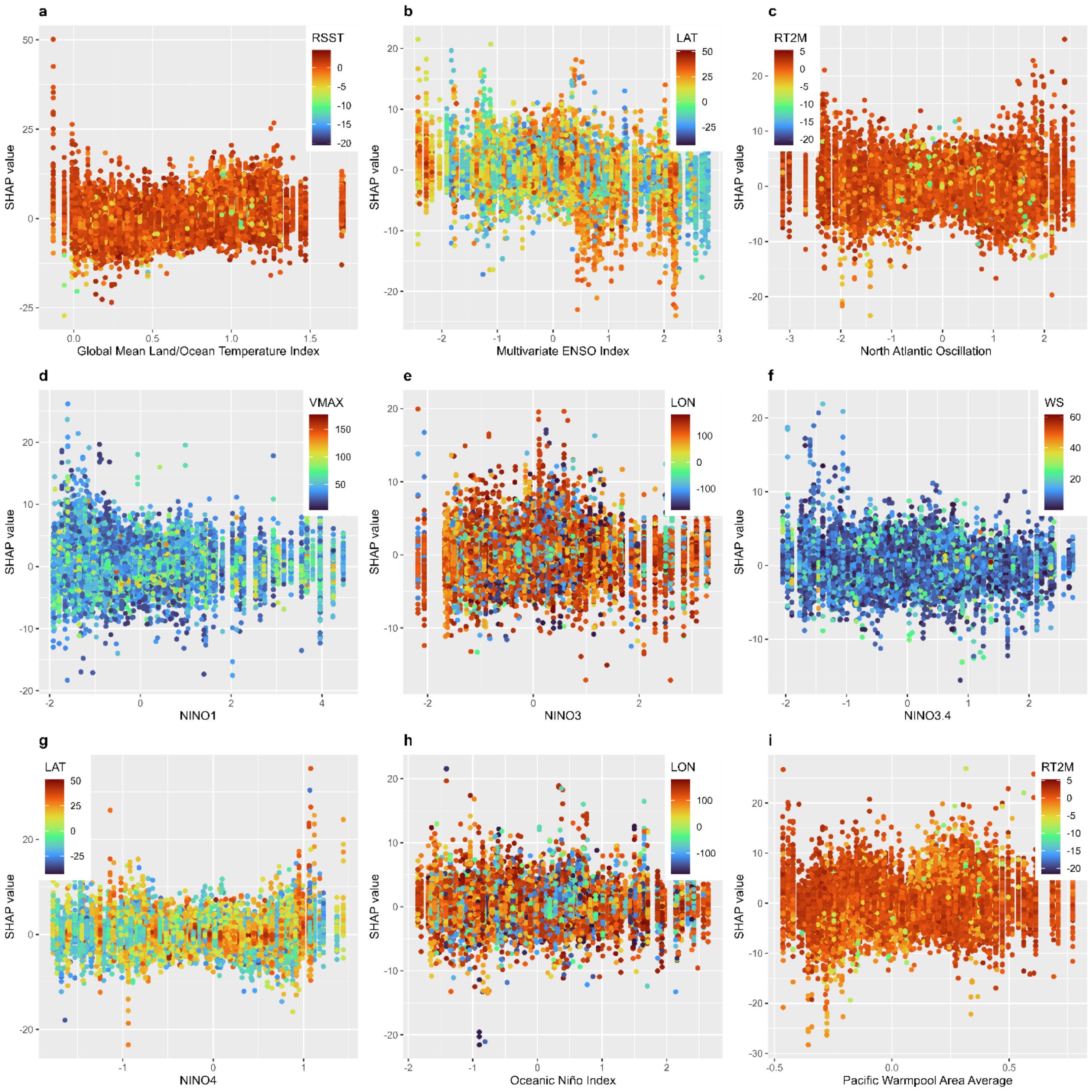

Supplementary Fig. 5. Relationship between participating features and DIST30 SHAP value for the global XGBoost model (PART 3).

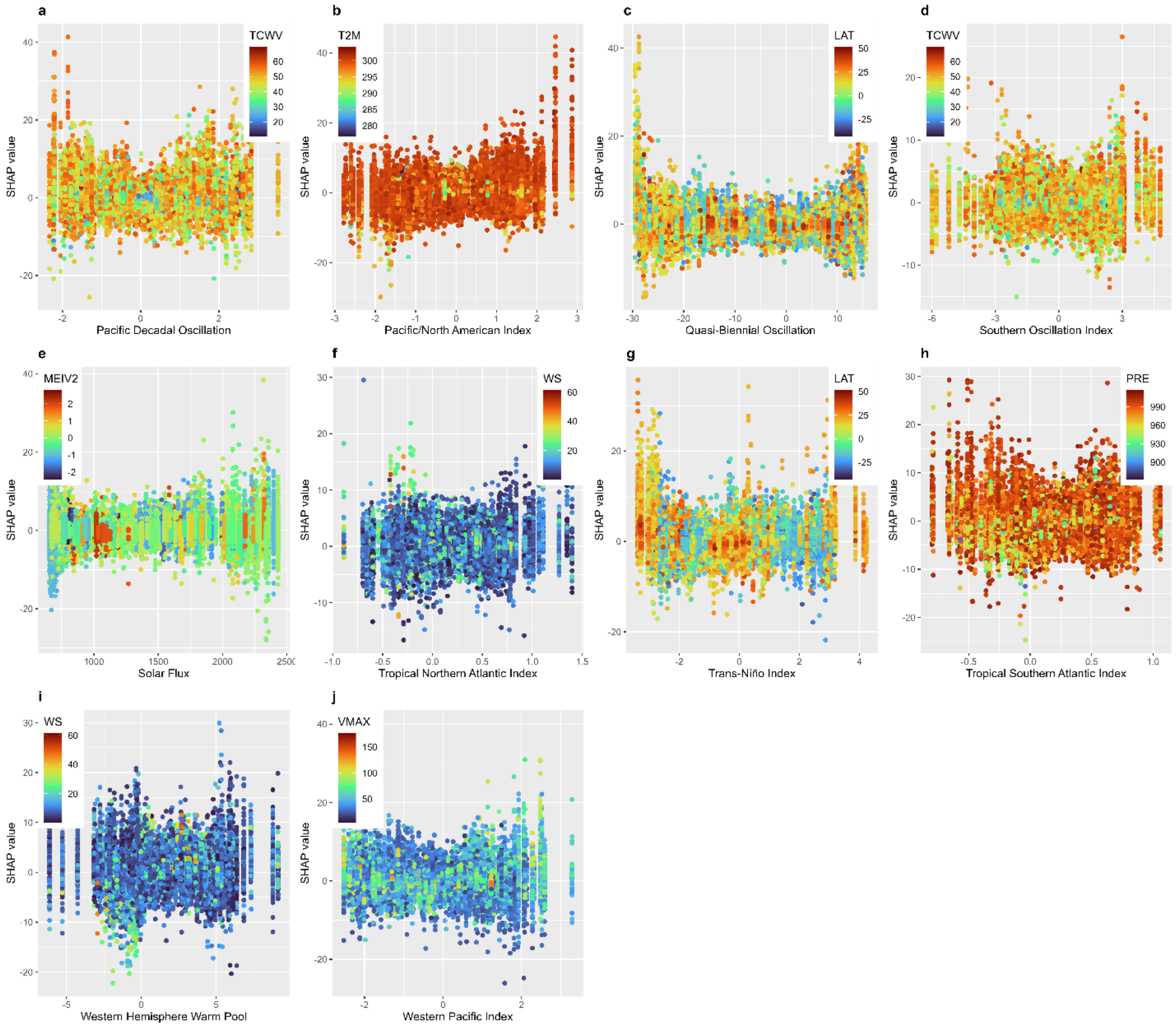

Supplementary Fig. 6. Relationship between participating features and DIST30 SHAP value for the global XGBoost model (PART 4).

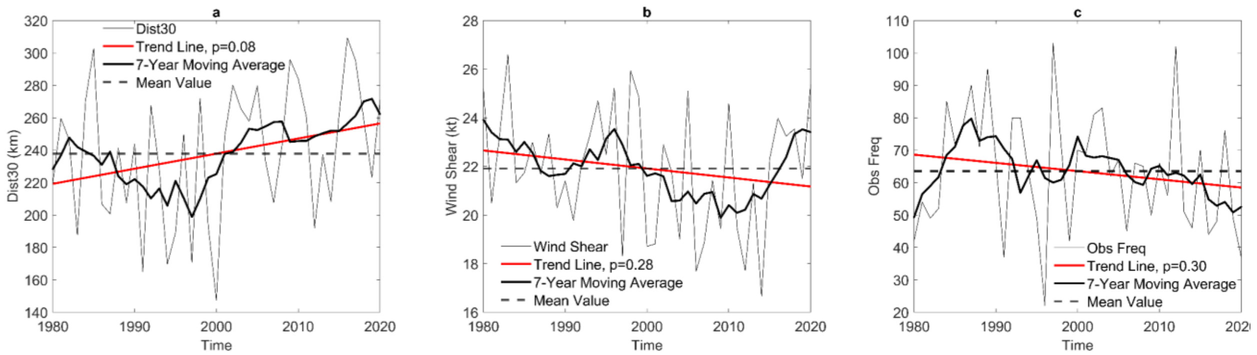

Supplementary Fig. 7. Interpreting the trend in distance at the threshold of 30 mm/3 hours (DIST30). a. the changes of DIST30 in higher latitudes of southern hemisphere (< -25°S); b. the changes of wind shear < -25°S; c. the changes of observation frequency < -25°S.

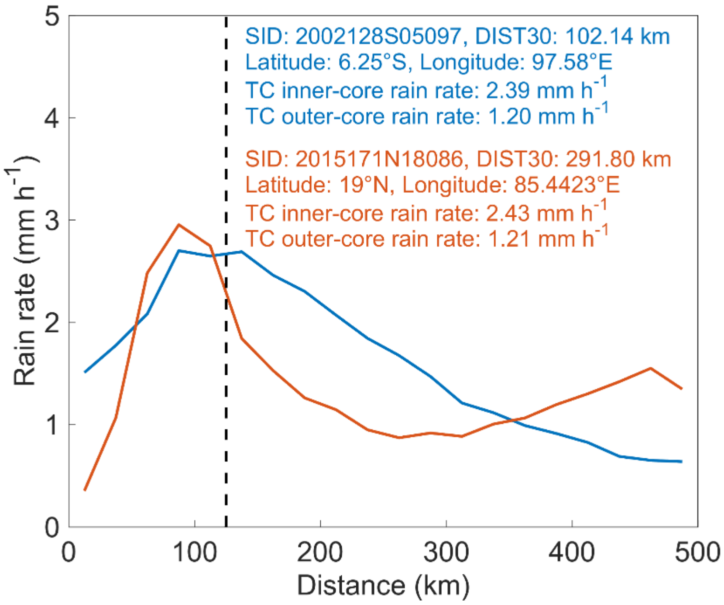

Supplementary Fig. 8. The distance series of TC rain rate. The profiles have similar precipitation in the inner core and outer regions, but differ in their value of DIST30 due to the different precipitation structure of TC.
